# Rapid evidence review of harm reduction interventions and messaging for people who inject drugs during pandemic events: implications for the ongoing COVID-19 response

**DOI:** 10.1186/s12954-020-00445-5

**Published:** 2020-12-01

**Authors:** Rebecca Wilkinson, Lindsey Hines, Adam Holland, Sema Mandal, Emily Phipps

**Affiliations:** 1 Rebecca Wilkinson, Public Health Registrar, School of Primary Care, Population Sciences and Medical Education, Southampton General Hospital, University of Southampton, Tremona Road, Southampton, SO16 6YD UK; 2 Lindsey Hines, Population Health Sciences, Bristol Medical School, University of Bristol, Bristol, UK; 3 Adam Holland Population Health Sciences, Bristol Medical School, University of Bristol, Bristol, UK; 4 Sema Mandal Blood Safety, Hepatitis, STI and HIV Division, National Infection Service, Public Health England, London, UK; 5 Emily Phipps Blood Safety, Hepatitis, STI and HIV Division, National Infection Service, Public Health England, London, UK

**Keywords:** Harm reduction, People who inject drugs (PWID), Pandemic, COVID-19

## Abstract

**Background:**

People who inject drugs are at increased health risk in a pandemic due to their greater susceptibility to severe disease and as a consequence of the restrictions put in place to halt the spread of infection. Harm reduction (HR) services, which aim to reduce the negative effects of drug use on health, are likely to be diminished in a pandemic. However, innovative HR interventions and messaging may also develop in response to such a crisis. It is vital to understand the most effective ways to deliver HR in pandemic situations so that guidance can be provided for current and future disruptions to service provision.

**Methods:**

A rapid evidence review was conducted with the aim of exploring what HR interventions and messaging are most effective during a pandemic-type situation. Ten health databases were systematically searched using terms relevant to the research aim. A search was also made of grey literature, including a targeted search of HR messaging from key national and service provider websites.

**Results:**

In the initial search, 121 pieces of evidence were identified which, after screening and de-duplication, resulted in 60 for inclusion. The included evidence consists mainly of non-peer reviewed, pre-publication or expert opinion pieces. The rapid findings suggest that HR services should be deemed essential during a pandemic, with staff supported to work safely and social distancing adaptations implemented. Services should be encouraged to operate more flexibly; for instance, in deciding the amounts of take-home supplies of injecting equipment and medications. The evidence on HR communication was very limited but key messages on infection control, uncertain drug supply and accessing services were identified.

**Conclusions:**

This rapid evidence review identifies implications for national policy makers, commissioners and HR service providers. A person-centred rather than disease-centred approach to HR delivered by collaborating partners, as well as prioritizing tailored HR messaging, is recommended. Further research evaluating the delivery of HR services and messaging, particularly focusing on health inequalities, is urgently needed.

## Background

A novel coronavirus, severe acute respiratory syndrome coronavirus 2, which causes coronavirus disease 2019 (COVID-19), was isolated in December 2019 and declared as a global pandemic by the World Health Organization (WHO) in March 2020 [1]. The impact of COVID-19 and the measures required to combat it are disproportionately felt by society’s most vulnerable populations. People who inject drugs (PWID) are likely to have a number of characteristics that make them more vulnerable, such as living on low incomes and in poor quality, crowded situations where social distancing is difficult. In addition, PWID are more likely to have physical and mental health comorbidities that may render them at increased risk of infection, more severe disease and worse outcomes [2].

Harm reduction (HR) can be defined as a public health approach which prioritizes reducing the negative effects of drug use rather than eliminating it or attaining abstinence [3]. HR interventions include needle and syringe programmes (NSP), opioid substitution therapy (OST) and provision of naloxone as an emergency antidote to opiate overdose.

During the COVID-19 pandemic, many HR services were reduced or suspended completely in order to redeploy staff or facilitate the social distancing required to curb the spread of infection. However, in some areas innovative HR interventions and messaging were developed in response to the crisis. The impact of the pandemic, and these associated service changes, on PWID is not yet known but it is likely that there will have been a worsening of outcomes [2, 4, 5]. Therefore, it is vital that lessons are learnt quickly about the most effective ways to deliver HR in situations such as the COVID-19 pandemic so that guidance can be provided for the ongoing COVID-19 response and similar outbreaks.

This report presents preliminary findings from a rapid review of the available evidence (up until 14/08/2020). The aim of the review was to identify which HR interventions and messages for PWID are most effective during situations such as a global pandemic.

## Methods

This rapid evidence review was undertaken at speed to address the urgent need for evidence and guidance on delivery of HR services and messaging for PWID during a pandemic.

The review protocol was registered with PROSPERO [6].

Databases searched between 10 and 14 August 2020 were PROSPERO, Cochrane, TRIP, Medline, PsycInfo, Web of Science, EMBASE, PubMed, OpenGrey, PLOS and Google Scholar, and Table 1 summarizes the search terms used (full details of the searches are included in Appendix 1—see Additional file 1).

**Table 1:** Summary of search terms

Population	P* who inject drugs PWID* Substance misuse* Inject* drug use* Substance abuse*
Intervention	Harm reduct* Harm min* Needle syringe Provi* Injecting Equipment Provi* Opiate Substitution Therapy
Situation	Service disrupt* lockdown COVID* Coronavirus SARS* Pandemic Big event*

*indicates truncation

Broad inclusion criteria were used as the scoping searches indicated a lack of peer-reviewed studies. Thus, grey literature, pre-publication and non-peer reviewed papers were also included as well as evidence suggested by experts in the field. The searches were, however, restricted to English language only.

The scoping searches revealed a lack of evidence on messaging specifically directed at PWID, so a targeted search (via Google) was conducted for COVID-19-related HR messaging from key national public health bodies and drug service providers.

The evidence was screened initially (RW) on whether the title alone appeared to have relevance to the research aim. The second screen (RW and AH) was based on abstract or summary and used the inclusion criteria detailed in Table 2. The search was not restricted to studies with a comparison group because of the lack of formal evaluations/studies.

**Table 2:** Inclusion criteria used for second screen

Factor	Criteria
Population	PWID
Intervention	Any HR intervention
Situation	Similar to COVID-19 pandemic

Data were extracted into a spreadsheet recording study bibliographic details, HR intervention details and key findings relating to the research aim.

As part of the rapid review approach, evidence was not subject to a robust quality appraisal but details of the type of evidence, study design and expert affiliation were extracted to allow a limited assessment of quality.

A narrative synthesis approach was used to collate and describe the key findings from the evidence in relation to the research aim.

## Results

### Overview of included studies

In total, 121 pieces of evidence were identified, these included 106 from the systematic searches plus a further 15 from citation searching and the targeted search for HR messaging. After de-duplication, 86 pieces of evidence were entered into the second round of screening.

Twenty-six pieces of evidence were excluded after abstract screening (see Table 2), leaving 60 pieces of evidence identified as appropriate for inclusion in the review. The majority (n = 20) of these exclusions were due to no information about HR interventions. Appendix 2 outlines the key details of the included studies (see Additional file 2).

Of the 60 pieces of evidence identified, 48% (n = 29) were expert opinion pieces (of which most were academic experts).

There were also 11 studies (mainly case reports, descriptive or qualitative). In instances where multiple reports were available from the same study, this was included as one piece of evidence.

There were four non-peer reviewed evidence reviews included.

The review covered 16 pieces of grey literature classified as guidance, which included COVID-19-related HR messaging for PWID. This type of messaging was not always directly available on Government websites but they often sign-posted to messaging produced by reputable non-Governmental organizations (NGOs), think-tanks or service providers so these have been included.

Based on the types of evidence included in the review, the quality would be considered low-moderate.

Table 3 shows the range of countries that the pieces of evidence were from.

**Table 3:** Included evidence by country

Country	Evidence (*n* = 60)
Canada and USA	25
International (i.e., more than one continent)	10
UK	14
Other Europe	6
Other	5

### Narrative synthesis

#### Designating HR services as essential

Designating HR services as essential, so that they are not suspended in the event of a pandemic-type scenario, has been recommended by many experts [7, 8] and globally recognized bodies, including the International Society of Addiction Medicine (ISAM) [9], the International Network of People who Use Drugs (INPUD) [10] and the European Monitoring Centre for Drugs and Drug Addiction (EMCDDA) [2]. Continued unrestricted access to HR services, such as NSP [9], could be facilitated by ensuring an adequate supply of personal protective equipment (PPE) for staff [7, 11, 12]. There is no clear consensus, however, on which HR interventions to prioritize when staff or resources are limited.

By designating HR as essential, these services can not only sustain their vital work but also offer additional functions [13]. For instance, as PWID are likely to be more vulnerable to any pandemic disease, and the impacts of their substance misuse may mask or mimic disease symptoms, frequent screening for the pandemic infection within HR services is recommended [9, 14]. Additionally, supplies of sanitizing materials can be included within HR packs [14, 15].

An initiative to provide ‘essential journey’ cards, that PWID may use when collecting medications during lockdown to demonstrate their journey is essential, is yet to be evaluated but has been recommended by some authors [16, 17].

Continued access to blood-borne virus (BBV) testing and treatment for PWID is vital to identify cases and reduce transmission [13]; innovative ways to deliver this, such as rapid testing, need to be identified [14].

#### Developing emergency plans

Emergency preparedness of HR services needs improving [22]. This may include developing contingency plans, such as for periods of equipment or medication shortage, which detail how HR services will be maintained (e.g., outreach, home delivery, virtual/phone consultations) [2, 11, 15]. The Larney and Bruneau (2020) review of the impact of ‘big events’ on substance misuse services emphasized the importance of emergency planning and reported that they found no publications describing how HR and drug treatment service providers should prepare for an emergency [19].

#### Adjusting HR services to comply with social distancing

Numerous papers have recommended ways in which HR services can remain open and comply with social distancing [20] such as through adjusting patient flow [11] or mechanical segregation [21]; the methods suggested for specific HR interventions are detailed below.

#### NSP

In terms of NSP, various alternatives to conventional collection are possible. For instance, a UK study concluded that home delivery, provision by post, peer-supported distribution and vending machines should be considered [22]. The authors of the UK study acknowledge that vending machines may be challenging to get in place quickly because of their sourcing and installation. They also point out that there are already direct postal sales of injecting equipment to some people who inject image and performance enhancing drugs so establishing free postal needle/syringe provision to all PWID could be relatively easily achieved by utilizing existing delivery services [22]. However, evidence of how such services should be coordinated and associated governance considerations has not yet been established.

Other authors also recommend vending machines, which provide 24-h access, and no-contact collection [7, 8]. The LUCID-B study in Bristol reported positive feedback from PWID in relation to home delivery because it meant they did not have to travel, it kept them safe from COVID-19 and it prevented re-use of equipment [23].

The Larney and Bruneau (2020) review of evidence from other ‘big events’ concluded that, in a pandemic-type scenario, NSP should offer as many needles and syringes to clients as requested and that flexible NSP, such as mobile or outreach models, will increase access [19]. Other authors have also recommended this ‘low threshold’ approach (as opposed to one-for-one exchange) to needle/syringe provision [7, 24].

#### OST

Similar to NSP, outreach and home delivery have been recommended for OST in a pandemic-type situation [15, 25]. Flexibility for services to relax supervision and increase take home doses has also been suggested [8, 12, 13, 15, 21]. Many authors state that these changes should be based on the stability of the patient, with highest risk patients still able to access the clinic [9, 16, 26–28]. Clearly there are inherent risks in this approach but methods, such as the use of technology (e.g., “smart” pill bottles/lock boxes that dispense doses on a remotely set timescale), would mitigate them [29].

This review found examples of new models of delivery of OST services. For instance, in Rhode Island USA, regulatory changes meant that initiation of OST by telephone could be developed; whilst the authors are positive about this approach, it has not yet been evaluated [30].

In Ireland, a model of remote care has been developed which begins with an assessment of COVID-19 risk by telephone followed by a single-patient visit to local services to provide a point of care drug screen and complete necessary documentation. Contact episodes are maintained through remote video assessment and ongoing management by a primary care addiction specialist. This model is yet to be evaluated but it appears to offer lower COVID-19 transmission risks, increased access to OST and reduced waiting times [31].

Much of the evidence makes recommendations around telehealth [14, 25, 32, 33] as well as describing other technology that HR services could utilize, including smartphone and web-based interventions, text messaging for continuing contact and care, machine learning and wearable devices, including digital phenotyping and ecological momentary assessment, biofeedback and virtual reality [34, 35]. Lead time and availability of technology (for both services and clients) will limit implementation of these options in the short term with text-messaging, smartphone and web-based interventions being the most simple and quick to roll-out.

One USA telehealth provider, Bicycle Health, has reported how it adapted its services to respond to COVID-19 guidance, such as urine testing via video link [36]. However, others have highlighted potential problems with these virtual solutions, such as the patient not having access to a private space for the call [29], and the risk of exacerbating inequalities which is considered further below.

Several authors suggested buprenorphine as a safer take home option than methadone [29, 31, 37] and providing it as depot (long-acting injection) was recommended [8, 38].

#### Naloxone

There is conflict between social distancing and the physically, socially and emotionally intimate nature of injecting drug use [39]; for instance, naloxone, as a HR intervention, relies on social connections. Several authors have suggested, that during a pandemic-type situation, naloxone should be made more accessible (with appropriate patient and family education) because of the increased overdose risk resulting from using drugs alone when socially distancing and due to uncertain supply [29, 35].

One author suggests virtual injection supervision, which allows individuals to inject in the presence of an observer on the internet who is prepared to intervene in the event of an overdose or virtual peer support which also uses the internet to make social support available to PWID at a physical distance [39]. Alternatively a USA study of NSP changes during COVID-19 recommended scheduling a phone check-in after use for people who are using alone [14].

#### Safe supply

Several authors have suggested ‘safe supply’ (defined as a legal and regulated supply of drugs that traditionally have been accessible only through the illicit drug market) could provide a solution to the conflict between social distancing and HR [40, 41]. One piece of evidence describes an unpublished study which found those receiving prescription alternatives to illicit drugs are able to avoid more routine contacts with drug dealers and can reduce activities that might put them at risk of acquiring or transmitting pandemic infections (e.g., sex work); however, the authors acknowledge that a full evaluation is needed [42].

#### Role of Pharmacies

Many authors highlighted the important role of pharmacies in delivering HR interventions during a pandemic-type situation if other services become unavailable [2, 43–45]. However, at such times, pharmacies may have reduced opening hours [31]. PWID who participated in the LUCID-B study reported finding the long queues for pharmacies at the start of lockdown very off-putting [23].

### Holistic approach

Much of the evidence suggested that HR needs to be part of a holistic approach to supporting PWID during a pandemic-type situation. For instance, several authors have emphasized the need for enhanced mental health support for PWID, with video or internet-based psychotherapy and phone counseling generally recommended [9, 11, 18, 19, 38].

Messages about how to access healthcare have been recommended as PWID may no longer have opportunistic access to treatment service staff and, therefore, may miss discussing wider physical health issues [23].

Links to social and economic services were also emphasized as important. For instance, ‘Housing First’ was highlighted as an approach which can facilitate social distancing and provide stability for PWID to engage in HR and manage extended OST take home doses appropriately [33, 34]. With increased duration of take home OST, one review concluded that accommodation for the homeless should have capacity for the safe storage of medications and space to designate as a safer use room [38].

### Inequalities

A pandemic-type scenario has the potential to exacerbate health inequalities already experienced by PWID, such as increased morbidity and mortality and reduced service provision [33]. Additionally, much of the evidence indicates a risk of widening inequalities within the injecting drug population by moving to interventions that require PWID to have access to a particular level of technology to be able to engage [29, 32, 34, 35, 39]. Providing mobile phones to clients [23, 29] or using peers to engage with the most marginalized PWID in their community [22, 24] are suggested strategies to mitigate this risk.

Overall, in the available evidence, there was little consideration of sub-groups within the population who inject drugs; one Ukrainian study considered older people as a sub-group of PWID finding that they need social support to engage in care [46] while another piece of evidence suggested needles/syringes should be provided by home delivery for PWID living in non-urban areas [7].

The Larney and Bruneau (2020) review found few studies considered the impact of ‘big events’ specifically on women meaning there is limited evidence to inform women-specific and gender-sensitive COVID-19 responses for women who use drugs. They suggest this is important because women who use drugs are vulnerable to gender-based violence, and scarcity of drugs is likely to exacerbate conflict and risks of exploitation and/or victimization [19].

### HR messaging

Developing a communications plan, at individual service level, is suggested in the literature [15]. Other authors stress that HR information materials should be made inclusive by ensuring they are suitable for various cultures, available in different languages/formats [16] and distributed through multiple new channels of communication, such as mobile apps, peer networks and social media sites [16, 23, 26].

Due to the lack of evidence on HR messaging, a search was done of the communications provided during the COVID-19 pandemic by key national bodies and service providers. With the exception of the USA [47], Government websites did not tend to provide direct messaging for PWID but they did sign-post to other independent resources which have been included in this review.

Important HR messages identified in this review can be grouped under the themes of infection control, and uncertain drug supply as detailed below: -

### Messaging relating to infection control

Most of the messaging considered in this review included COVID-19-related hygiene advice, such as hand washing and cleaning surfaces [47–54]. This was also highlighted by a group of Canadian experts [15] and many other authors stated the importance of using HR services to educate about infection control measures [2, 8, 14, 24, 33, 38].

Cleaning the packages that drugs are supplied in (e.g., with alcohol wipes) was covered in some messaging [49, 51, 52] and expert opinion [15]. Not carrying drugs packages in the body (e.g., mouth, rectum, vagina) was also mentioned [49, 52, 54].

Advice for PWID to prepare drugs themselves and avoid sharing equipment was common to almost all the messaging included in this review and was backed up with guidance around stocking up on supplies of equipment (such as needles and syringes) [47–49, 49–56]. Most authors suggested at least two weeks [48, 51, 52, 54] supply of equipment but some recommended 3–4 weeks’ worth [49, 53]. Some messaging went on to include advice on how to clean syringes in the event of running out of supplies [4, 47, 51, 53].

A Canadian expert group also suggest that public health messages around self-isolation and social distancing should be modified for people who use drugs, who live in shelters or who are involved in sex work [15]. Some of the messaging included in the review did specifically provide advice for sex workers to limit close contact [48, 53].

The USA Government guidance explicitly recommends that PWID make use of the other services offered by NSP such as testing for BBV [47] while the WHO suggests HR messaging should be used to dispel myths that substance use somehow protects a person from infection [12].

### Messaging relating to uncertain drug supply

An international group of experts recommended that messaging needs to include information about overdose risks associated with changes in the quantity and quality of the drugs market [24] and most of the messaging included in this review did cover this. For instance, advice for PWID included using a test dose, or small amount, initially to see how it makes them feel [47, 48, 51, 55]. Some messaging advised stocking up on drugs/having a reserve in case of shortages [49, 53, 54] with others warning about the legality and dangers of obtaining large amounts of drugs [48, 52]. One USA study also concluded that educating participants on the increased risks of overdose through supply disruptions should include advice to cautiously increase personal supply in the event of a shortage [14].

Most messaging followed on from explaining the supply issue with advice about reducing the risk of overdose by accessing naloxone or by making a plan with family or friends to check in after using drugs [47–51, 53–56].

Guidance on managing involuntary withdrawal, such as stocking up on medications to relieve symptoms, was mentioned in much of the messaging because of the risk of drugs being in short supply or PWID not being able to access them due to self-isolation [48–54].

One of the UK service providers advises PWID to consider snorting rather than injecting during the pandemic as it is less risky when the quality of supply is unknown. They also suggest administering doses slowly when injecting to allow the drug to take effect, in order to reduce the likelihood of accidental overdose [55].

## Discussion

This rapid review found 60 pieces of evidence relating to HR interventions and messaging in a pandemic-type situation and, through narrative synthesis, has identified the following implications for HR services, HR communication and further research.

### Implications for HR services

HR services should prepare for future pandemics by developing emergency plans and involving relevant PWID advocacy groups in redesigning services. This review focused on interventions so found little evidence on the role of operational partnerships, and working with organizations who support PWID, but these would be important for the implementation of, and may have implications on effectiveness of, interventions and messaging.

Government and commissioning organizations should ensure that HR services are designated as essential and take action to facilitate maintained provision (e.g., by providing appropriate PPE). There is no consensus from the evidence on which HR interventions to prioritize so local areas should base this on the needs of their population. As essential services, HR providers could take on additional functions such as screening clients for the pandemic infection and testing for BBV.

To ensure equitable access, all NSP should be modified to enable social distancing (e.g., delivery rather than collection) and should have a low threshold for supply of injecting equipment.

A key message from the evidence is that complying with social distancing requires a more flexible approach to OST. This could be in terms of relaxing supervision and increasing take-home doses as well as considering more innovative ways of delivering OST services (such as telehealth and other technology). However, action must be taken to mitigate possible widening of inequalities; this might include provision of mobile phones to clients or targeted use of peer supporters.

Due to an increased risk of overdose [43], access to naloxone is particularly important during a situation such as a global pandemic; therefore, services should employ innovative strategies to overcome the conflict with social distancing (such as through virtual support) and work with clients to identify suitable contacts (i.e., from their household or ‘bubble’).

Strong partnerships between HR services and other services (particularly pharmacies, housing providers and physical and mental health care services) are needed in order to offer a holistic approach. For instance, HR services should work with homeless accommodation providers to ensure they can provide safe storage of medications and space for a safer use room.

Evidence from an international group of experts concludes that consultation with PWID advocacy groups is needed when designing HR services that can respond to pandemic-type situations [58].

Looking to the future, several authors suggest that the COVID-19 pandemic response has provided an opportunity for much needed change in the delivery of HR services [34, 35, 44]. Innovations and regulatory changes, such as flexible OST dosing and dispensing, made during the COVID-19 pandemic need to be evaluated and, if effective in a non-pandemic situation, sustained into the future [10, 31, 44, 45, 59].

### Implications for HR communications

This rapid review found limited evidence on HR messaging during a pandemic-type situation but by including a targeted search of HR messaging from key national and service provider websites the following implications have been identified.

HR services should develop a communications plan, which uses multiple channels, to employ in the ongoing COVID-19 response or future pandemic-type situations. However, relaying HR messages to PWID during a pandemic is so vitally important that government websites should include messaging directly for PWID and not rely on signposting to service providers or NGOs.

**Fig. 1:**
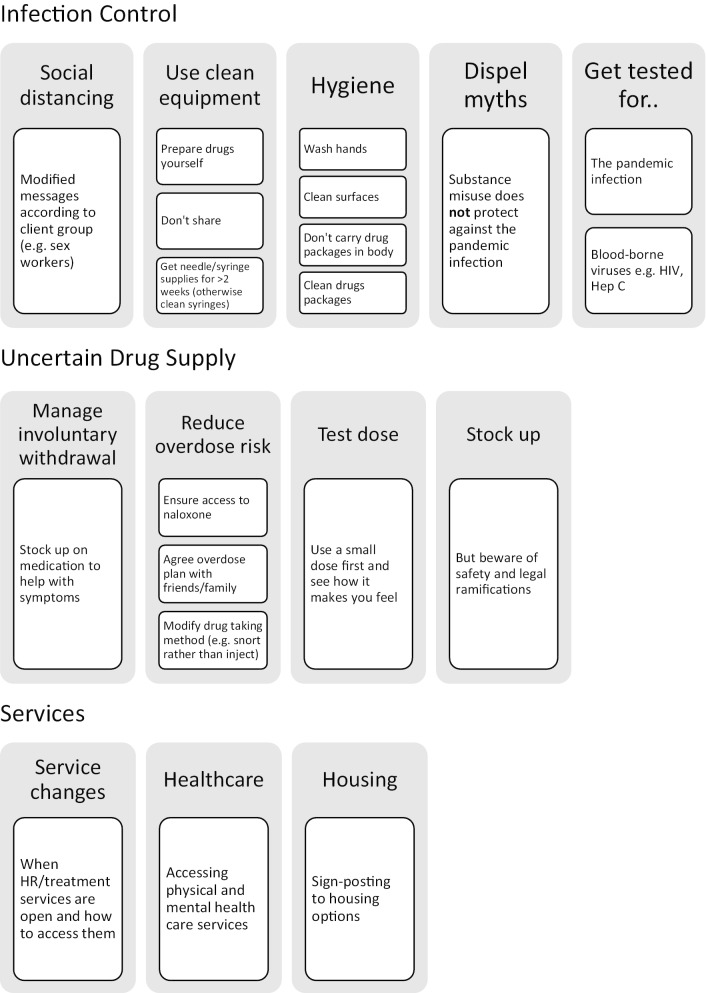
Summary of HR messaging during a pandemic situation

HR messaging should include advice on issues of infection control and uncertain drug supply, as well as key information about accessing HR and other services to fit with a holistic approach; these messages are summarized in Fig. 1.

### Further research

To meet an urgent need for evidence and guidance, this rapid review did not include a systematic quality assessment of the evidence but a brief appraisal revealed most was non-peer reviewed, pre-publication or expert opinion and so would be considered of low-medium quality. Thus, further research to evaluate the innovations and changes to HR services and messaging during the COVID-19 pandemic is urgently needed. This is particularly important as many experts feel that these changes should be sustained into the post-pandemic era so the impacts, both intended and unintended, require robust assessment. Additionally, the majority of evidence is opiate focussed, which likely reflects the main cohort accessing services, but suggests a need for further focussed work with non-opiate clients.

This conclusion agrees with that of a rapid review conducted by the National Collaborating Centre for Methods and Tools in Canada, in that little evidence is available on the effectiveness of strategies to mitigate the harmful effects of illicit drug use during the COVID-19 pandemic [60]. Specifically, evidence around HR messaging during a pandemic-type situation is particularly lacking.

### Limitations

As previously stated, this rapid review presents preliminary findings of an emerging evidence base. It only includes evidence available until 14/08/2020. The COVID-19 pandemic is still ongoing and new evidence is becoming available all the time. Further robust systematic reviews are needed to confirm the findings as the evidence base expands.

Searches were restricted to English language evidence only which is likely to have excluded useful evidence from areas such as Eastern Europe. In fact, much of the evidence was from the USA and Canada so is likely to be most applicable to higher-income countries.

Time and resource constraints also meant that most of the searches, screening and data extraction was done by just one member of the review team. While this ensured consistency, it does risk bias in what evidence was included and excluded.

In response to a lack of evidence on HR messaging, a targeted approach was taken to find additional messaging aimed directly at PWID from key national and services provider websites based on author knowledge of relevant national stakeholders. A further detailed, systematic search of the grey literature may glean more results, as would exploration of emerging channels of communication (such as apps) which were not explicitly investigated in this review.

## Conclusions

Preliminary findings from this rapid review suggest that HR services should be considered as essential during a pandemic and should be encouraged to operate flexibly to best meet the needs of their local community of PWID. Additionally, working with partners to offer holistic client-centered support is crucial.

The evidence on HR communication during a pandemic is scarce but the targeted search has identified key messages around infection control, uncertain drug supply and accessing services which should be delivered via multiple channels.

Of overarching importance is awareness of, and action to mitigate, exacerbation of inequalities through over-reliance on new remote and technology-based service delivery which may exclude the most marginalized.

## Supplementary information

**Additional file 1.** Details of searches including database, search terms, number of hits and number eligible after first screen.

**Additional file 2.** Key details of the included evidence such as author, title, source, type of evidence and a description of the harm reduction intervention or messaging covered.

## Data Availability

Not applicable.

## Abbreviations

BBV: Blood-borne viruses
COVID-19: Coronavirus disease 2019
EMCDDA: European Monitoring Centre for Drugs and Drug Addiction
HR: Harm reduction
INPUD: International Network of People who Use Drugs
ISAM: International Society of Addiction Medicine
LUCID-B: Living Under Coronavirus and Injecting Drugs in Bristol
NGOs: Non-Governmental organizations
NSP: Needle and syringe programmes
OST: Opioid substitution therapy
PPE: Personal protective equipment
PWID: People who inject drugs
WHO: World Health Organization
