# Supplementary material for: Rapid evidence review of harm reduction interventions and messaging for people who inject drugs during pandemic events: implications for the ongoing COVID-19 response
Source: Harm Reduct J. 2020 Dec 1;17:95. doi: 10.1186/s12954-020-00445-5 (PMC7705852; doi:10.1186/s12954-020-00445-5)
Supplement: Supplementary file 1 — Additional file 1. Details of searches including database, search terms, number of hits and number eligible after first screen. [file 12954_2020_445_MOESM1_ESM.docx]

**Appendix 1: Details of Searches**

| **Date** | **Database** | **Search terms** | **No. of hits** | **No. eligible after initial screen** |
| --- | --- | --- | --- | --- |
| 10/08/2020 | PROSPERO | ("Harm reduct*" OR "Harm min*" OR "Needle syringe Provi*" OR "Injecting Equipment Provi*" OR "Opiate Substitution Therapy") AND ("P* who inject drugs" OR "PWID*" OR "Substance misuse*" OR "Inject* drug use*" OR "Substance abuse*") AND ("Service disrupt*" OR "lockdown" OR "COVID*" OR "Coronavirus" OR "SARS*" OR "Pandemic") | 16 | 1 |
| 10/08/2020 | Cochrane | "Harm reduct*" OR "Harm min*" OR "Needle syringe Provi*" OR "Injecting Equipment Provi*" OR "Opiate Substitution Therapy" in Title Abstract Keyword AND "P* who inject drugs" OR "PWID*" OR "Substance misuse* OR "Inject* drug use*" OR "Substance abuse*"" in Title Abstract Keyword AND "Service disrupt*" OR "lockdown" OR "COVID*" OR "Coronavirus" OR "SARS*" OR "Pandemic" in Title Abstract Keyword - (Word variations have been searched) | 1 | 0 |
| 11/08/2020 | TRIP | (inject* drugs) AND (harm red*) AND (COVID OR coronavirus) | 33 | 3 |
| 10/08/2020 | Medline EBSCO | ("Harm reduct*" OR "Harm min*" OR "Needle syringe Provi*" OR "Injecting Equipment Provi*" OR "Opiate Substitution Therapy") AND ("P* who inject drugs" OR "PWID*" OR "Substance misuse*" OR "Inject* drug use*" OR "Substance abuse*") AND ("Service disrupt*" OR "lockdown" OR "COVID*" OR "Coronavirus" OR "SARS*" OR "Pandemic") | 38 | 12 |
| 11/08/2020 | PsycInfo EBSCO | ("Harm reduct*" OR "Harm min*" OR "Needle syringe Provi*" OR "Injecting Equipment Provi*" OR "Opiate Substitution Therapy") AND ("P* who inject drugs" OR "PWID*" OR "Substance misuse*" OR "Inject* drug use*" OR "Substance abuse*") AND ("Service disrupt*" OR "lockdown" OR "COVID*" OR "Coronavirus" OR "SARS*" OR "Pandemic") | 24 | 3 |
| 12/08/2020 | Web of Science | (corona virus OR coronavirus OR covid* OR lockdown OR pandemic OR sars* OR service disrupt*) AND (harm min* OR harm reduct* OR injecting equipment provi* OR needle syringe provi* OR opiate substitution therapy) AND (injecting drug use* OR pwid* OR substance abuse* OR substance misuse* OR who inject drugs) | 26 | 6 |
| 12/08/2020 | EMBASE Ovid | (corona virus OR coronavirus OR covid* OR lockdown OR pandemic OR sars* OR service disrupt*) AND (harm min* OR harm reduct* OR injecting equipment provi* OR needle syringe provi* OR opiate substitution therapy) AND (injecting drug use* OR pwid* OR substance abuse* OR substance misuse* OR who inject drugs) | 18 | 6 |
| 12/08/2020 | PubMed | COVID* AND substance misuse | 15 | 6 |
| 12/08/2020 | PubMed | (corona virus OR coronavirus OR covid* OR lockdown OR pandemic OR sars* OR service disrupt*) AND (harm min* OR harm reduct* OR injecting equipment provi* OR needle syringe provi* OR opiate substitution therapy) AND (injecting drug use* OR pwid* OR substance abuse* OR substance misuse* OR who inject drugs) | 45 | 15 |
| 12/08/2020 | OpenGrey | Various keywords including COVID* AND substance misuse | 0 | 0 |
| 12/08/2020 | PLOS | Various keywords including COVID* AND substance misuse | 0 | 0 |
| 13/08/2020 | Google Scholar | COVID substance misuse (N.B Sorted by relevance - I looked through until had 5 pages with no relevant results - that was page 30). | 6,450 | 42 |
| 13/08/2020 | Google Scholar | COVID harm minimisation injecting drug | 17,400 | 6 |
| 13/08/2020 | Medline EBSCO | MH Harm Reduction AND MH Drug Misuse AND MH Coronavirus | 0 | 0 |
| 14/08/2020 | Google Scholar | COVID substance abuse | 23,300 | 6 |
| 14/08/2020 | Google | Specific searches for COVID-19 related HR messaging from key national public health bodies and drug service providers | N/A | 12 |
