# Supplementary material for: Rapid evidence review of harm reduction interventions and messaging for people who inject drugs during pandemic events: implications for the ongoing COVID-19 response
Source: Harm Reduct J. 2020 Dec 1;17:95. doi: 10.1186/s12954-020-00445-5 (PMC7705852; doi:10.1186/s12954-020-00445-5)
Supplement: Supplementary file 2 — Additional file 2. Key details of the included evidence such as author, title, source, type of evidence and a description of the harm reduction intervention or messaging covered. [file 12954_2020_445_MOESM2_ESM.docx]

**Appendix 2: Key details of the included evidence**

| **Ref no** | **Search** | **Authors** | **Name of evidence** | **Date** | **Source** | **Hyperlink** | **Type of evidence** | **If a study, what is the Research Design/ Methodology?** | **If expert opinion, what type of experts?** | **Country** | **Details of HR intervention or messaging** |
| --- | --- | --- | --- | --- | --- | --- | --- | --- | --- | --- | --- |
| 2 | Google Scholar | A Schlosser, S Harris | Care during COVID-19: Drug use, harm reduction, and intimacy during a global pandemic | Jul-20 | Int J of Drug Policy | <https://www.ncbi.nlm.nih.gov/pmc/articles/PMC7392208/> | Expert opinion |  | Academics | US | Virtual injection supervision where individuals to inject in the presence of an observer on the internet who is prepared to intervene in the event of an overdose. |
| 4 | Google Scholar | Ali Farhoudian et al | COVID-19 and Substance Use Disorders: Recommendations to a Comprehensive Healthcare Response. An International Society of Addiction Medicine (ISAM) Practice and Policy Interest Group Position Paper | Apr-20 | Autonomic Neuroscience: Basic & Clinical | <https://discovery.dundee.ac.uk/files/49005620/COVID_ISAM_PPIG_Position_Paper.pdf> | Expert opinion |  | Academics, Treatment providers | International | Messaging COVID screening NSP OST |
| 5 | Google Scholar | Amira Guirguis | There is a vulnerable group we must not leave behind in our response to COVID-19: people who are dependent on illicit drugs | Apr-20 | The Pharmaceutical Journal | <https://www.pharmaceutical-journal.com/20207926.article?utm_campaign=2482_PJ_daily_alert&utm_medium=email&utm_source=Pharmaceutical%20Journal&firstPass=false> | Expert opinion |  | Academic | UK | Messaging OST |
| 6 | Google Scholar | B Conway, D Truong, K Wuerth | COVID-19 in homeless populations: unique challenges and opportunities | Jun-20 | FUTURE VIROLOGY | <https://www.futuremedicine.com/doi/full/10.2217/fvl-2020-0156> | Expert opinion |  | Treatment providers | Canada | OST |
| 7 | Google Scholar | Basu D, Ghosh A, Subodh B N, Mattoo S K. | Opioid substitution therapy with buprenorphine-naloxone during COVID-19 outbreak in India: Sharing our experience and interim standard operating procedure. | May-20 | Indian J Psychiatry | <http://www.indianjpsychiatry.org/article.asp?issn=0019-5545;year=2020;volume=62;issue=3;spage=322;epage=326;aulast=Basu> | Guidance |  |  | India | Opioid substitution therapy with buprenorphine-naloxone |
| 8 | Google Scholar | Crowley, D; Delargy, I | A national model of remote care for assessing and providing opioid agonist treatment during the COVID-19 pandemic: a report | Jul-20 | HARM REDUCTION JOURNAL | <https://link.springer.com/article/10.1186/s12954-020-00394-z> | Study | Case study |  | Ireland | A remote model for assessment and ongoing care of OAT patients |
| 9 | Google Scholar | D Mongan, B Galvin, L Farragher, M Dunne, M Nelson | Impact of COVID-19 on drug services in four countries. An evidence brief | Jun-20 | Health Research Board, Ireland | <https://www.drugsandalcohol.ie/32296/1/HRB_evidence%20brief%20for%20DPU%20Covid-19%20rapid%20assessment_June_2020.pdf> | Evidence Review |  |  | Irish paper but based on areas in 4 different countries - New South Wales, Scotland, New York State and British Columbia | COVID messaging OST MH support e.g. suicide assessments |
| 11 | Google Scholar | IW Holloway, A Spaulding, A Miyashita | COVID‐19 vulnerability among people who use drugs: recommendations for global public health programmes and policies | Jun-20 | Int J of the AIDS Society | <https://onlinelibrary.wiley.com/doi/full/10.1002/jia2.25551> | Expert opinion |  | Academics | US | Telehealth for OST |
| 12 | Google Scholar | J Chang, J Agliata, M Guarinieri | COVID-19 - Enacting a ‘new normal’ for people who use drugs | Jul-20 | International Journal of Drug Policy | <https://www.sciencedirect.com/science/article/pii/S0955395920301730> | Expert opinion |  | NGO | International | OST NSP Drug consumption rooms |
| 13 | Google Scholar | Jemberie, W et al. | Substance Use Disorders and COVID-19: Multi-Faceted Problems Which Require Multi-Pronged Solutions | 2020 | Frontiers in Psychiatry | <https://www.diva-portal.org/smash/get/diva2:1456567/FULLTEXT01.pdf> | Expert opinion |  | Academics | International | Telehealth Take home OST |
| 14 | Google Scholar | Jiang H, Su H, Zhang C, et al. | Challenges of methadone maintenance treatment during the COVID-19 epidemic in China: Policy and service recommendations. | 2020 | Eur Neuropsychopharmacol. | <https://www.ncbi.nlm.nih.gov/pmc/articles/PMC7152875/> | Study | Descriptive |  | China | methadone maintenance treatment |
| 15 | Google Scholar | KM Peavy, J Darnton, P Grekin, M Russo, CJB Green | Rapid Implementation of Service Delivery Changes to Mitigate COVID‑19 and Maintain Access to Methadone Among Persons with and at High‑Risk for HIV in an Opioid Treatment Program | Apr-20 | AIDS Behav | <https://link.springer.com/content/pdf/10.1007/s10461-020-02887-1.pdf> | Study | Descriptive |  | US | OST |
| 17 | Google Scholar | López-Pelayo, H., Aubin, H., Drummond, C. et al. | The post-COVID era”: challenges in the treatment of substance use disorder (SUD) after the pandemic. | Jul-20 | BMC Med | <https://bmcmedicine.biomedcentral.com/articles/10.1186/s12916-020-01693-9#citeas> | Expert opinion |  | Academics | International | Telehealth  OST Housing First |
| 18 | Google Scholar | M Karamouzian, C Johnson, T Kerr | Public health messaging and harm reduction in the time of COVID-19 | May-20 | The Lancet Psychiatry | <https://www.thelancet.com/journals/lanpsy/article/PIIS2215-0366(20)30144-9/fulltext#articleInformation> | Expert opinion |  | Academics | Canada | Messaging NSP Emergency planning for harm reduction OST Naloxone |
| 20 | Google Scholar | M Tyndall | Safer opioid distribution in response to the COVID-19 pandemic | Jul-20 | International Journal of Drug Policy | <https://www.sciencedirect.com/science/article/pii/S095539592030219X> | Expert opinion |  | Academics | Canada | OST Naloxone Safe supply |
| 22 | Google Scholar | Mark Whitfield, Howard Reed, Jane Webster, Vivian Hope | The impact of COVID-19 restrictions on needle and syringe programme provision and coverage in England | Jul-20 | Int J of Drug Policy | <https://www.sciencedirect.com/science/article/pii/S0955395920301912> | Study | Descriptive |  | UK | NSP (home delivery, provision by post, peers supported distribution, vending machines) |
| 23 | Google Scholar | Marsden, J., Darke, S., Hall, W. et al. | Mitigating and learning from the impact of COVID‐19 infection on addictive disorders. | 2020 | Addiction. | <http://eprints.whiterose.ac.uk/160151/1/add.15080.pdf> | Expert opinion |  | Academics | International | OST take home |
| 29 | Google Scholar | S Chiappini, A Guirguis, A John, JM Corkery | COVID-19: The Hidden Impact on Mental Health and Drug Addiction | Jul-20 | Front. Psychiatry | <https://www.frontiersin.org/articles/10.3389/fpsyt.2020.00767/full> | Expert opinion |  | Academics | UK | COVID precautions Telehealth OST |
| 30 | Google Scholar | Sara N. Glick  et al | The Impact of COVID‑19 on Syringe Services Programs in the United States | Apr-20 | AIDS and Behavior | <https://link.springer.com/content/pdf/10.1007/s10461-020-02886-2.pdf> | Study | Mixed methods assessment |  | US | NSP  Naloxone |
| 31 | Google Scholar | Sokol, Randi et al | Guidance for Treating Patients with Opioid Use Disorder (OUD) with buprenorphine- Naloxone (B/N) in the COVID-19 Era via Telehealth: A Review of Previous Evidence, New COVID-19 OUD Treatment Guidelines, and a Case Report of their Application | Aug-20 | Annals of Family Medicine | <https://deepblue.lib.umich.edu/bitstream/handle/2027.42/155566/Sokol%20article%20file.pdf?sequence=1&isAllowed=y> | Study | Evidence review and case report |  | US | OST |
| 32 | Google Scholar | Sun Y, Bao Y, Kosten T, Strang J, Shi J, Lu L. | Editorial: Challenges to Opioid Use Disorders During COVID-19. | 2020 | Am J Addict. | <https://www.ncbi.nlm.nih.gov/pmc/articles/PMC7262307/> | Expert opinion |  | Academics | International | OST |
| 33 | Google Scholar | TC Green, J Bratberg, DS Finnell | Opioid use disorder and the COVID 19 pandemic: A call to sustain regulatory easements and further expand access to treatment | Apr-20 | Substance Abuse | <https://www.tandfonline.com/doi/full/10.1080/08897077.2020.1752351> | Expert opinion |  | Academics | US | OST Naloxone |
| 34 | Google Scholar | TS Bartholomew, N Nakamura, LR Metsch | Syringe services program (SSP) operational changes during the COVID-19 global outbreak | Jun-20 | Int J of Drug Policy | <https://www.ncbi.nlm.nih.gov/pmc/articles/PMC7290194/> | Study | Descriptive  Survey |  | US | NSP |
| 35 | Google Scholar | WC Becker, DA Fiellin | When Epidemics Collide: Coronavirus Disease 2019 (COVID-19) and the Opioid Crisis | Jul-20 | Annals of Internal Medicine | <https://www.acpjournals.org/doi/full/10.7326/M20-1210> | Expert opinion |  | Academics | US | OST |
| 36 | Google Scholar | WHO | Substance use considerations during #COVID19 | 2020 | WHO | <https://apps.who.int/iris/bitstream/handle/10665/333463/Subst-use%20-COVID19-eng.pdf> | Guidance |  |  | International |  |
| 38 | Google Scholar | YC Leong, PR Verbeek | Does intranasal naloxone administration increase the risk of 2019 coronavirus disease transmission? | May-20 | Canadian Journal of Emergency Medicine | <https://www.cambridge.org/core/journals/canadian-journal-of-emergency-medicine/article/does-intranasal-naloxone-administration-increase-the-risk-of-covid19-transmission/F4114C24D56BF64355E9C48222A99960> | Expert opinion |  | Academics, Treatment providers | Canada | Naloxone |
| 39 | Google Scholar | Zaami Simona, Marinelli Enrico, Varì Maria Rosaria | New Trends of Substance Abuse During COVID-19 Pandemic: An International Perspective | 2020 | Frontiers in Psychiatry | <https://www.frontiersin.org/articles/10.3389/fpsyt.2020.00700/full> | Expert opinion |  | Academics | Italy | OST |
| 40 | Prospero | Sarah Larney et al | Rapid review of the impacts of Big Events on drug-related risks and harms, and delivery of harm reduction and drug treatment services: implications for responding to COVID-19 | 08/05/2020 |  | <https://cihr-irsc.gc.ca/e/52044.html> | Evidence Review | Evidence review |  | Canada | Reducing overdose risk NSPs Mental health |
| 42 | PubMed | Armitage R, Nellums LB. | Substance misuse during COVID-19: protecting people who use drugs | 15/05/2020 | Public Health | <https://www.ncbi.nlm.nih.gov/pmc/articles/PMC7218358/> | Expert opinion |  | Academics | UK | Messaging |
| 43 | PubMed | Chayama KL, Ng C, McNeil R. | Calls for Access to Safe Injecting Supplies as a Critical Public Health Measure During the COVID-19 Pandemic | 10/08/2020 | J Addict Med | <https://pubmed.ncbi.nlm.nih.gov/32769778/> | Expert opinion |  | Academics | Canada and US | NSP |
| 46 | PubMed | Dunlop A, et al | Challenges in maintaining treatment services for people who use drugs during the COVID-19 pandemic | 08/05/2020 | Harm Reduct J | <https://www.ncbi.nlm.nih.gov/pmc/articles/pmid/32375887/> | Expert opinion |  | Academics, Treatment providers | Australia | OST Naloxone NSP |
| 50 | PubMed | Heimer R, McNeil R, Vlahov D. | A Community Responds to the COVID-19 Pandemic: a Case Study in Protecting the Health and Human Rights of People Who Use Drugs | 29/07/2020 | J Urban Health | <https://www.ncbi.nlm.nih.gov/pmc/articles/PMC7384769/> | Study | Case study |  | US | OST |
| 51 | PubMed | Henry BF et al | COVID-19, mental health, and opioid use disorder: Old and new public health crises intertwine | 20/06/2020 | Psychol Trauma | <https://content.apa.org/fulltext/2020-43095-001.html> | Expert opinion |  | Academics | US |  |
| 53 | PubMed | Jacka BP, Phipps E, Marshall BDL. | Drug use during a pandemic: Convergent risk of novel coronavirus and invasive bacterial and viral infections among people who use drugs | 04/08/2020 | Int J Drug Policy | <https://www.ncbi.nlm.nih.gov/pmc/articles/PMC7388892/> | Expert opinion |  | Academics, Government | US and UK | NSP OST Messaging |
| 54 | PubMed | Karimi-Sari H et al. | Harm reduction during the COVID-19 outbreak in Iran | 27/07/2020 | Lancet Psychiatry | <https://www.ncbi.nlm.nih.gov/pmc/articles/PMC7377766/> | Study | Qualitative |  | Iran | NSP OST |
| 57 | PubMed | Rozanova J et al | Social Support is Key to Retention in Care during Covid-19 Pandemic among Older People with HIV and Substance Use Disorders in Ukraine | 16/07/2020 | Subst Use Misuse | <https://pubmed.ncbi.nlm.nih.gov/32666857/> | Study | Qualitative |  | Ukraine | OST |
| 58 | PubMed | Samuels EA et al | Innovation During COVID-19: Improving Addiction Treatment Access | 15/05/2020 | J Addict Med | <https://www.ncbi.nlm.nih.gov/pmc/articles/PMC7236851/> | Expert opinion |  | Academics, Treatment providers | US | Telehealth for OST initiation |
| 62 | PubMed | Vasylyeva TI, Smyrnov P, Strathdee S, Friedman SR. | Challenges posed by COVID-19 to people who inject drugs and lessons from other outbreaks | 23/07/2020 | J Int AIDS Soc | <https://www.ncbi.nlm.nih.gov/pmc/articles/PMC7375066/> | Expert opinion |  | Academics | International |  |
| 63 | TRIP | National Collaborating Centre for Methods and Tools. | Rapid Review: What is the effect of the COVID-19 pandemic on opioid and substance use and related harms? | June 19, 2020 | The National Collaborating Centre for Methods and Tools, McCaster University | <https://www.nccmt.ca/uploads/media/media/0001/02/675c5ceeffc452bcea887967aa0e4ce8eed6d16e.pdf> | Evidence Review |  |  | Canada | OST Naloxone Sanitisation NSP |
| 64 | TRIP | Nicole Andruszkiewicz & David Gogolishvili | Possible benefits of providing safe supply of substances to people who use drugs during public health emergencies such as the COVID-19 pandemic. | Apr-20 | Ontario HIV Treatment Network | <http://www.ohtn.on.ca/rapid-response-possible-benefits-of-providing-safe-supply-of-substances-to-people-who-use-drugs-during-public-health-emergencies-such-as-the-covid-19-pandemic/> | Evidence Review |  |  | Canada | Safe supply |
| 66 | Expert advice | ARC West | Understanding the experiences of people who inject drugs during the COVID-19 pandemic |  |  | <https://arc-w.nihr.ac.uk/research/projects/understanding-the-experiences-of-people-who-inject-drugs-during-the-covid-19-pandemic/> | Study | Qualitative |  | UK | NSP OST Messaging |
| 72 | Google Scholar | SE Wakeman, TC Green, J Rich | An overdose surge will compound the COVID-19 pandemic if urgent action is not taken | May-20 | Nature Medicine | <https://www.nature.com/articles/s41591-020-0898-0> | Expert opinion | N/A | Academics | US | Discusses facilitating provision for OST, NSP and naloxone |
| 73 | Google Scholar | J Du, N Fan, M Zhao, W Hao, T Liu, L Lu, J Shi | Expert consensus on the prevention and treatment of substance use and addictive behaviour-related disorders during the COVID-19 pandemic | Jul-20 | General Psychiatry | <https://www.ncbi.nlm.nih.gov/pmc/articles/PMC7358097/> | Expert opinion | N/A | Academic, Treatment providers | China | Discussion of advice to be offered to PWUD, their families and services |
| 74 | Google Scholar | PA Spagnolo, C Montemitro | New Challenges in Addiction Medicine: COVID-19 Infection in Patients With Alcohol and Substance Use Disorders—The Perfect Storm | Jul-20 | Am J of Psychiatry | <https://ajp.psychiatryonline.org/doi/full/10.1176/appi.ajp.2020.20040417> | Expert opinion | N/A | Academics | US | Discussion of risks faced by PWUD(&A) and considerations around treatment provision |
| 75 | Google Scholar | IE Leppla, MS Gross | Optimizing Medication Treatment of Opioid Use Disorder During COVID-19 (SARS-CoV-2) | May-20 | Journal of Addiction Medicine | <https://www.ncbi.nlm.nih.gov/pmc/articles/PMC7273937/> | Expert opinion | N/A | Academics | US | Drug treatment / OST |
| 77 | Citation searching | European Monitoring Centre for Drugs and Drug Addiction | The implications of COVID-19 for people who use drugs (PWUD) and drug service providers | Mar-20 |  | <https://www.emcdda.europa.eu/publications/topic-overviews/covid-19-and-people-who-use-drugs_en> | Guidance |  |  | Europe | OST |
| 78 | Citation searching | Conference of the Chairmen of Quality Assurance Commissions of the Associations of Statutory Health Insurance Physicians in Germany | Information on opioid substitution and Sars-CoV-2/Covid-19 Advice for physicians | Mar-20 |  | <https://www.forum-substitutionspraxis.de/images/Germany_Information_on_opioid_substitution_and_Sars-CoV-2Covid-19_-Advice_for_physicians.pdf> | Guidance |  |  | Germany | OST |

| 79 | Citation searching | Paxton Bach, Samantha Robinson, Christy Sutherland, and Rupinder Brar | Innovative strategies to support physical distancing among individuals with active addiction | May-20 | Lancet Psychiatry | <https://www.ncbi.nlm.nih.gov/pmc/articles/PMC7255226/> | Expert opinion |  | Academics | Canada | Safe supply |
| --- | --- | --- | --- | --- | --- | --- | --- | --- | --- | --- | --- |
| 80 | Google | Change Grow Live (CGL) | Coronavirus advice for people who use drugs | May-20 |  | <https://www.changegrowlive.org/advice-info/coronavirus/drugs-harm-reduction-advice> | Guidance |  | Provider | England | Messaging |
| 81 | Google | Turning Point | Information on using drugs more safely |  |  | <https://www.turning-point.co.uk/covid19-information-for-service-users> | Guidance |  | Provider | England | Messaging |
| 82 | Google | We are with you | How to use drugs more safely during coronavirus |  |  | <https://www.wearewithyou.org.uk/help-and-advice/use-drugs-safely-coronavirus/> | Guidance |  | Provider | England | Messaging |
| 84 | Google | Inclusion | Staying safer during coronavirus |  |  | <https://indd.adobe.com/view/42c433ba-f79e-4b1d-83ef-1b2dd757e954> | Guidance |  | Provider | England | Messaging |
| 85 | Google | CDC | COVID-19 Questions and Answers: For People Who Use Drugs or Have Substance Use Disorder |  |  | <https://www.cdc.gov/coronavirus/2019-ncov/need-extra-precautions/other-at-risk-populations/people-who-use-drugs/QA.html> | Guidance |  | Gov | US | Messaging |
| 86 | Google | Canadian Drugs Policy Coalition | COVID-19: Advice for People Who Use Drugs | Mar-20 |  | <https://drugpolicy.ca/covid-19-advice-for-people-who-use-drugs/> | Guidance |  | Think tank | Canada | Messaging |
| 87 | Google | Welsh Government | Coronavirus (COVID-19): guidance for substance misuse and homelessness services | May-20 |  | <https://gov.wales/coronavirus-covid-19-guidance-for-substance-misuse-and-homelessness-services-html> | Guidance |  | Gov | Wales | Messaging |
| 88 | Sign-posting | Scottish Drugs Forum | DO YOU INJECT DRUGS? | Mar-20 | Link from Scottish Gov website | <http://www.sdf.org.uk/wp-content/uploads/2020/03/COVID-19-Drug-injecting-A5-Flyer-Digital.pdf> | Guidance |  | Think tank | Scotland | Messaging |
| 89 | Sign-posting | Groundswell | Coronavirus advice for people who use drugs | May-20 | Link from EMCDDA website | <https://groundswell.org.uk/wp-content/uploads/2020/05/Drugs-and-COVID-19-Action-Update-V2-19.05.20-.pdf> | Guidance |  | NGO | UK | Messaging |
| 90 | Google | Public Health England | COVID-19: guidance for commissioners and providers of services for people who use drugs or alcohol | Jul-20 |  | [https://www.gov.uk/government/publications/covid-19-guidance-for-commissioners-and-providers-of-services-for-people-who-use-drugs-or-alcohol/](https://www.gov.uk/government/publications/covid-19-guidance-for-commissioners-and-providers-of-services-for-people-who-use-drugs-or-alcohol/covid-19-guidance-for-commissioners-and-providers-of-services-for-people-who-use-drugs-or-alcohol#considerations-for-people-using-drugs-or-alcohol) | Guidance |  | Gov | UK | Messaging |
| 91 | Sign-posting | FRANK | Coronavirus: drinking and taking drugs during easing of the lockdown | Jul-20 | Link from PHE website | <https://www.talktofrank.com/news/easing-lockdown-tips> | Guidance |  | NGO | UK | Messaging |
| 92 | Sign-posting | Harm Reduction Coalition | Safer Drug Use During the COVID-19 Outbreak | Mar-20 | Link from Irish Health Research Board website | <https://www.drugsandalcohol.ie/31756/2/COVID19-safer-drug-use-1.pdf> | Guidance |  | NGO | Ireland | Messaging |
